# ForageGrassBase: molecular resource for the forage grass meadow fescue (*Festuca pratensis* Huds.)

**DOI:** 10.1093/database/baaa046

**Published:** 2020-06-15

**Authors:** Jeevan Karloss Antony Samy, Odd Arne Rognli, Mallikarjuna Rao Kovi

**Affiliations:** Department of Plant Sciences (IPV), Faculty of Biosciences (BIOVIT), Norwegian University of Life Sciences (NMBU), Ås, Akershus 1432, Norway

## Abstract

Meadow fescue (*Festuca pratensis* Huds.) is one of the most important forage grasses in temperate regions. It is a diploid (2n = 14) outbreeding species that belongs to the genus *Festuca*. Together with *Lolium perenne*, they are the most important genera of forage grasses. Meadow fescue has very high quality of yield with good winter survival and persistency. However, extensive genomic resources for meadow fescue have not become available so far. To address this lack of comprehensive publicly available datasets, we have developed functionally annotated draft genome sequences of two meadow fescue genotypes, ‘HF7/2’ and ‘B14/16’, and constructed the platform ForageGrassBase, available at http://foragegrass.org/, for data visualization, download and querying. This is the first open-access platform that provides extensive genomic resources related to this forage grass species. The current database provides the most up-to-date draft genome sequence along with structural and functional annotations for genes that can be accessed using Genome Browser (GBrowse), along with comparative genomic alignments to *Arabidopsis*, *L. perenne*, barley, rice, *Brachypodium* and maize genomes. We have integrated homologous search tool BLAST also for the users to analyze their data. Combined, GBrowse, BLAST and downloadable data gives a user-friendly access to meadow fescue genomic resources. To our knowledge, ForageGrassBase is the first genome database dedicated to forage grasses. The current forage grass database provides valuable resources for a range of research fields related to meadow fescue and other forage crop species, as well as for plant research communities in general. The genome database can be accessed at http://foragegrass.org.

## Introduction

Grasslands cover 36% of the earth’s surface, and they are important as feed sources and pastures for livestock (1). Among several forage crops, meadow fescue is one of the most important forage grass species in temperate regions of the world (2).

**Figure 1:**
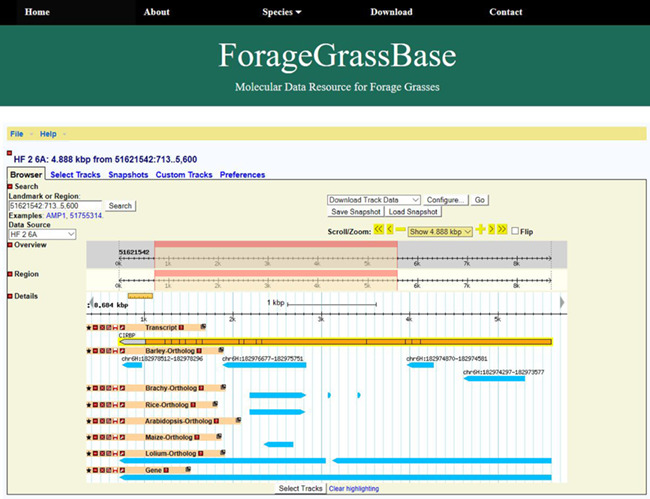
ForageGrassBase genome browser contains *F. pratensis* genome annotation and their orthologous regions in *Arabidopsis*, *Lolium perenne*, *Brachypodium*, barley, maize and rice.

Meadow fescues in general have better adaptations to the winter survival, whereas the closely related perennial ryegrass (*Lolium perenne* L.) has better nutritive value with high yield quality but lacks persistency and adaptation to winter survival. The *Lolium–Festuca* species complex is useful in plant breeding, since it is possible to make intergeneric hybrids (*Festulolium*) by combining *Lolium* and *Festuca* genomes (3). Thus, the complementation of traits in *Festulolium* hybrids for developing novel cultivars with improved quality and adaptation to winter survival is crucial for sustainable forage production. However, modest genomic resources have been developed for meadow fescue compared with other grass species like perennial ryegrass (4, 5).

In order to develop molecular tools that might enhance the development of better *Festulolium* hybrids, we initiated and have now developed high-quality genomic resources for meadow fescue, taking advantage of the close comparative relationships with other grass species such as *Arabidopsis*, perennial ryegrass (5), barley (*Hordeum vulgare*), rice (*Oryza sativa*), *Brachypodium distachyon* and maize (*Zea mays*). This brings the published resources for meadow fescue up to the level available for other plant species in databases such as Gramene (http://www.gramene.org/), PlantGDB (http://www.plantgdb.org/), Oryzabase (https://shigen.nig.ac.jp/rice/oryzabase/), *Arabidopsis* genome database (https://www.arabidopsis.org/), *Medicago truncatula* genome database (http://www.medicagogenome.org/). Compared with the Gramene, more genetic resources like gene expression, annotation and comparative genomics available in databases specifically developed for individual plant species. Hence, we took initiative to develop forage grass base, dedicated only to forage grass genomics, where the researchers and breeders can readily get access to all the necessary information.

High-quality annotated *Festuca* genomes are now available. As a first step, the genome sequences and genome annotations for two meadow fescue genotypes are made available through ForageGrassBase (http://foragegrass.org). ForageGrassBase was developed to make these substantial amounts of genomic data accessible through visualizations and analytic tools in a common framework. Integrating resources for other forage grass species into ForageGrassBase is in progress and for new forage grass genomes, when they become available.

## Materials and Methods

Bootstrap (HTML, CSS), Javascript, PHP and Python were used to develop ForageGrassBase. The Generic Genome Browser (GBrowse) (6) and BLAST (7) were also installed. R packages are used for BLAST results visualizations. The database was organized in a similar way as we developed and described in SalmoBase (8).

**Figure 2:**
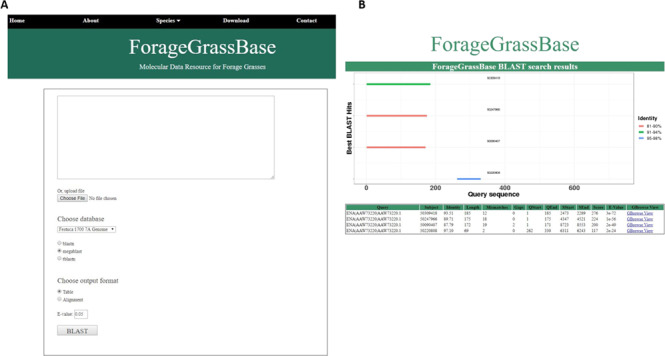
*Festuca pratensis* cultivars genome browsers with genome annotations and BLAST tool. (A) BLAST tool implemented to search for homologous regions in the reference genomes available. (B) BLAST results page shows the homologous regions.

*De novo* sequencing of the meadow fescue genomes were performed using Illumina mate pair sequencing and assembly was performed by the SOAPdenovo2 assembler (9). Furthermore, gene annotation was performed by in-house developed annotation pipelines and python scripts (Supplementary files). Briefly, Illumina reads were mapped to the assembly using STAR v2.3.1z12 (9). Cufflinks v2.2.180 (10) was used to assemble the reads into transcript models for all alignments. Gene models were tested by performing open reading frame (ORF) prediction using TransDecoder (https://github.com/TransDecoder/TransDecoder) using both pfamA and pfamB (11) databases for homology searches and a minimum length of 30 amino acids for ORFs without pfam support and BLASTP (12) analysis (evalue <1e-10) for all predicted proteins.

## Results and Discussion

### Genome browser

The GBrowse is simple and one of the most used genome browsers for visualization of genomes. We installed GBrowse to visualize and share genomic data of meadow fescue (Figure 1). Though two browsers are available for closely related perennial ryegrass genome (4, 5), the gene annotation and comparative genomics tracks are missing, and moreover, they are not integrated with other grass genomes. Currently, ForageGrassBase contains molecular data of two meadow fescue genotypes; *Festuca* HF2/7, a Norwegian genotype originating from a population selected for high frost tolerance and a Yugoslavian genotype, B14/16, which is used by our group to develop a mapping family for linkage map construction (13). Further, a comparative genome analysis was performed against other grass species like *Arabidopsis*, perennial ryegrass (5) barley, *Brachypodium*, rice and maize. These comparative genomics tracks consisting of gene names and chromosome positions were added to the genome browsers (Figure 1). More data and tracks will be added in the near future for other economically important forage grass species like timothy (*Phleum pratense*) to expand the forage grass genomics resources in ForageGrassBase.

### BLAST server

We have installed a BLAST server to search for homologous regions in the meadow fescue genome. Users having unknown sequences can use BLAST search to find the homologous regions in *Festuca* and their corresponding homologous genes and their physical location in *Arabidopsis*, perennial ryegrass, *Brachypodium*, barley, rice and maize (Figure 2A). After the search, our algorithm chooses the best hits and plots them in a unique way. Briefly, our BLAST output formatting algorithm combines all the hits for query sequence on each target, display horizontal bar for each hit based on the length of the hit and assigns color codes based on the similarity. In this way, it would be easier to interpret the results based on similarity and query coverage. BLAST results are connected to GBrowse, so the users can view the homologous regions and nearby genes and other genomic features in all these species (Figure 2B).

### Future plans and integrations

ForageGrassBase was developed based on high interest for the molecular data of meadow fescue. Genetic variations and gene expression data will be added using Genetic variation browser (GVBrowser) and Gene expression browser (GEBrowser) in the very near future. Due to rapid developments and lower costs of high-throughput sequencing technologies, we expect more forage grass genome sequence data to be available soon, and these resources and new tools will be added under ForageGrassBase.

### Database access and feedback

All the data used in developing this database are available through the ‘Download’ menu in ForageGrassBase. Genome sequences and gene annotation files for the two *Festuca* genotypes are available in ‘fasta’ and ‘gff3’ file formats to download and re-use. Users can send their questions and comments through ‘Contact form’ under ‘Contact’ menu.

## Conclusions

To the best of our knowledge, ForageGrassBase is the only online database to access, visualize and download data for the forage grass species meadow fescue and its homologous sequences/genes in rice, barley, *Brachypodium* and maize.

## Availability and requirements

ForageGrassBase can be accessed at https://foragegrass.org/.

## Availability of data and materials

This work does not contain additional data.

## Authors’ contributions

M.R.K., J.K.A.S. and O.A.R. conceived the idea of developing ForageGrassBase. O.A.R. and M.R.K. provided the genome sequences and annotation files. J.K.A.S. developed ForageGrassBase with inputs from M.R.K. and O.A.R. J.K.A.S. and M.R.K. wrote the manuscript and included comments from O.A.R. All authors read and approved the final manuscript.

## Consent to publish

Not applicable.

## Ethics approval and consent to participate

Not applicable.

## Supplementary Material

suppl_data_baaa046
